# The identification of metabolism-related subtypes and potential treatments for idiopathic pulmonary fibrosis

**DOI:** 10.3389/fphar.2023.1173961

**Published:** 2023-05-18

**Authors:** Changqing Yang, Guixin Wang, Wenyu Zhan, Yubao Wang, Jing Feng

**Affiliations:** ^1^ Respiratory Department, Tianjin Medical University General Hospital, Tianjin, China; ^2^ Tianjin Institute of Urology, Second Hospital of Tianjin Medical University, Tianjin, China; ^3^ Department of Urology, Second Hospital of Tianjin Medical University, Tianjin, China

**Keywords:** idiopathic pulmonary fibrosis, subtype, metabolism, prognosis, treatment, drug

## Abstract

**Background:** Idiopathic pulmonary fibrosis (IPF) is caused by aberrant repair because of alveolar epithelial injury and can only be effectively treated with several compounds. Several metabolism-related biomolecular processes were found to be involved in IPF. We aimed to identify IPF subtypes based on metabolism-related pathways and explore potential drugs for each subtype.

**Methods:** Gene profiles and clinical information were obtained from the Gene Expression Omnibus (GEO) database (GSE70867 and GSE93606). The enrichment scores for 41 metabolism-related pathways, immune cells, and immune pathways were calculated using the Gene Set Variation Analysis (GSVA) package. The ConsensusClusterPlus package was used to cluster samples. Novel modules and hub genes were identified using weighted correlation network analysis (WGCNA). Receiver operating characteristic (ROC) and calibration curves were plotted, and decision curve analysis (DCA) were performed to evaluate the model in the training and validation cohorts. A connectivity map was used as a drug probe.

**Results:** Two subtypes with significant differences in prognosis were identified based on the metabolism-related pathways. Subtype C1 had a poor prognosis, low metabolic levels, and a unique immune signature. CDS2, LCLAT1, GPD1L, AGPAT1, ALDH3A1, LAP3, ADH5, AHCYL2, and MDH1 were used to distinguish between the two subtypes. Finally, subtype-specific drugs, which can potentially treat IPF, were identified.

**Conclusion:** The aberrant activation of metabolism-related pathways contributes to differential prognoses in patients with IPF. Collectively, our findings provide novel mechanistic insights into subtyping IPF based on the metabolism-related pathway and potential treatments, which would help clinicians provide subtype-specific individualized therapeutic management to patients.

## 1 Introduction

Idiopathic pulmonary fibrosis (IPF) is a chronic respiratory disease characterized by the destruction of healthy pulmonary tissue, which is replaced by fibrotic remodeling (Richeldi et al., 2017). The incidence and mortality of IPF have increased worldwide, placing a considerable burden on society (Hutchinson et al., 2014; Maher et al., 2021). The prognosis of IPF is poor, with a 5-year survival rate of 31% (Khor et al., 2020) although some patients may live longer (Lederer and Martinez, 2018). Thus, there is an urgent need to identify the heterogeneity of IPF prognosis and develop precise therapies.

Metabolomics has recently become a popular topic for researchers exploring health conditions in humans (Surendran et al., 2022). Metabolomics has reportedly helped elucidate the pathological mechanism of IPF (Roque and Romero, 2021; Gonzalez-Garcia et al., 2022; Wygrecka et al., 2023). Several studies have demonstrated that lipidomic markers can be used to diagnose IPF, indicating their involvement in lipid metabolism (Yan et al., 2017; Rindlisbacher et al., 2018). Metabolic pathways related to energy consumption, such as the tricarboxylic acid cycle, are accelerated in the lungs of patients with IPF (Kang et al., 2016; Zhao et al., 2017). Similar results have been obtained in mouse models, demonstrating that metabolic pathways are involved in IPF pathogenesis (Xie et al., 2015; Chung et al., 2019). Based on these findings, researchers have explored possible treatments. Small molecule-mediated 8-oxoguanine DNA glycosylase-1 (OGG1) inhibition has a potential role in pulmonary fibrosis and a modulatory effect on metabolic syndromes (Tanner et al., 2023). Zhu et al. (2021) reported that drug-targeted iron metabolism could inactivate fibroblasts and attenuate pulmonary fibrosis. However, as a call for personalized management based on treatable traits (Amati et al., 2023), few studies have focused on distinguishing IPF to provide individualized metabolic therapies. Therefore, stratification of metabolic characteristics is potentially suitable for identifying the subtypes of candidate treatments for patients with IPF.

Accordingly, we aimed to classify patients into subtypes based on their metabolism-related pathways that significantly alter the prognosis. Furthermore, we investigated the hub genes to further aid in distinguishing between the two subtypes. Finally, putative drugs for precise treatment of different subtypes were probed.

## 2 Materials and methods

### 2.1 Data collection and processing

Gene expression data and related clinical information were extracted from the Gene Expression Omnibus (GEO) database (Supplementary Tables S1 and S2). The GSE70867 dataset was used for training analysis, and normalized gene profiles were mapped using the GPL14550 and GPL17077 probes. The GSE93606 dataset was used for validation analysis, and normalized gene profiles were mapped using GPL11532 probes. Patients in these datasets were diagnosed with IPF by matching their survival-related information. Survival status was defined as death as the positive endpoint and was censored as the negative endpoint. After removing the batch effect using the SVA R package (Johnson et al., 2007), the gene expression data were collected for further analysis. Subsequent analyses were performed using R version 4.1.3 and online tools.

### 2.2 Metabolism-related subtyping

Metabolism-related pathway gene sets were obtained using gene set enrichment analysis (GSEA) (Mootha et al., 2003; Subramanian et al., 2005). Single-sample GSEA (ssGSEA) was performed on the training and validation cohorts to calculate the enriched fraction of each pathway in the different samples using the GSVA R package (Barbie et al., 2009). A total of 41 metabolism-related pathways were used to construct a consistency matrix using the ConsensusClusterPlus R package (Wilkerson and Hayes, 2010). The PAM algorithm was selected to perform 100 bootstraps, each of which ensured an 80% involvement of the original dataset. The k-values of the clusters ranged from 2 to 6. After classification, the view was mapped using the uniform manifold approximation and projection (UMAP) method to reduce the space dimension. The Kaplan–Meier method was used to calculate the median survival time, and survival comparisons between different subtypes were performed using the log-rank test, with *p*-values <0.05 considered significant.

### 2.3 Immune-related analysis

Enriched fractions for immune pathways and cells were obtained for each sample using ssGSEA. The immune-related data were obtained from published literature (Charoentong et al., 2017). The Wilcoxon rank-sum test was used to assess the differences in immune cell infiltration and immune pathway enrichment between metabolism-related subtypes. Pearson’s correlation analysis was performed to visualize the relationships between the metabolic and immune pathways in each subtype.

### 2.4 Weighted gene co-expression network analysis

Weighted gene co-expression network analysis (WGCNA) (Langfelder and Horvath, 2008; 2012) was performed on 143 samples using 1,723 metabolism-related genes obtained from GSEA. An independence power value of approximately 0.85–0.9 was set to construct an unsigned topology matrix with a minimum of 30 genes and a maximum of 0.75 similarities between each module. Module–trait relationship analysis was then performed to calculate the correlation between each module and the features of the subtype. Module gene enrichment analysis was performed using Metascape (https://metascape.org/) (Zhou et al., 2019), and enrichment terms were significant at *p*-values <0.01. Hub genes were identified using Molecular Complex Detection (MCODE) (Bader and Hogue, 2003) in Metascape, and visualization was performed using Cytoscape (Shannon et al., 2003).

### 2.5 Diagnostic efficiency analysis of hub genes

Logistic regression was used to construct a model with hub genes to better predict the metabolism-related subtypes. A receiver operating characteristic (ROC) curve was constructed using the ROC R package (Sing et al., 2005) to assess the discriminatory ability of the model. A calibration curve was applied to assess the predictive accuracy of the model using the bootstrap method with 1,000 re-samplings (Van Calster et al., 2019). The Hosmer–Lemeshow (HL) test was added to the calibration curve, which recognized a *p*-value >0.05 as a good model fitting and calibration. The decision curve analysis (DCA) was applied to assess the clinical applicability of the model (Vickers and Elkin, 2006). The training and validation cohorts were subjected to these analyses.

### 2.6 Connectivity map analysis

To explore potential drugs for different subtypes, a Connectivity Map (CMap) (https://clue.io/) was used for drug identification. The L1000 method was used to identify perturbations in the mechanism of action (MoA) or biological functions caused by drug treatment of cells, which helped assess the possibility of drug application for different subtypes (Subramanian et al., 2017).

## 3 Results

### 3.1 The identification of the metabolism-related subtypes

**FIGURE 1:**
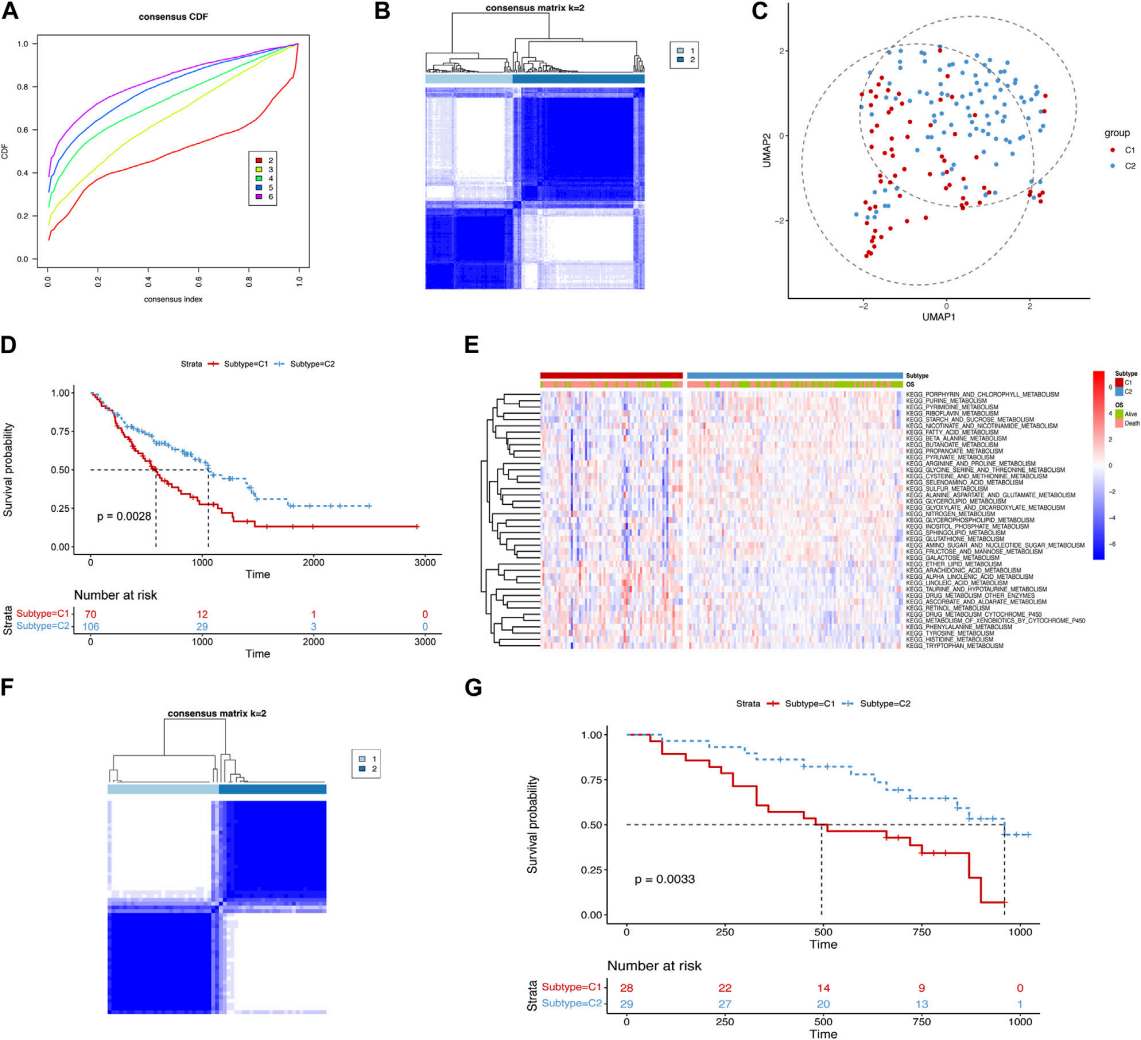
identification of metabolism-related subtypes of IPF. **(A)** Cumulative distribution function curves of the training cohort. **(B)** Sample clustering heatmap of the training cohort. **(C)** Uniform manifold approximation and projection (UMAP) plot for the two subtypes in the training cohort. **(D)** Kaplan–Meier curves displaying the overall survival of the two subtypes in the training cohort (median survival time of subtype C1: 586 days and C2: 1,055 days). **(E)** Heatmap of the differential enrichment scores of metabolism-related pathways for the two subtypes in the training cohort. **(F)** Sample clustering heatmap of the validation cohort. **(G)** Kaplan–Meier curves depicting the overall survival of the two subtypes in the validation cohort (median survival time of subtype C1: 495 days and C2: 960 days).

To better understand metabolism-related mechanisms in IPF, we calculated the scores of the metabolism-related pathways using ssGSEA in both the training and validation cohorts (Supplementary Tables S3 and S4). Based on the 41 metabolism-related pathways, we chose k = 2 to cluster the samples into two subtypes (Figures 1A,B). We then used UMAP to project the samples into a two-dimensional space, and subtype C1 was distinguished from C2 (Figure 1C). By constructing a survival analysis, we observed that subtype C1 had a significantly shorter survival time than C2 (Figure 1D). To provide an overview of the differences between the two subtypes, we generated a heatmap showing the landscape of the metabolism-related pathway enrichment (Figure 1E). Considering all enrichments, subtype C1 exhibited a lower degree of metabolism than C2. In the validation cohort, we used the same parameters to cluster the samples into two subtypes (Figure 1F). Consistent with the results in the training cohort, the metabolism-related subtypes were significantly different in the validation cohort (Figure 1G). These results indicate that metabolic heterogeneity exists in IPF, which manifests differently according to metabolic status and can consistently distinguish the prognosis of patients in different cohorts.

### 3.2 Immune analysis of metabolism-related subtypes

**FIGURE 2:**
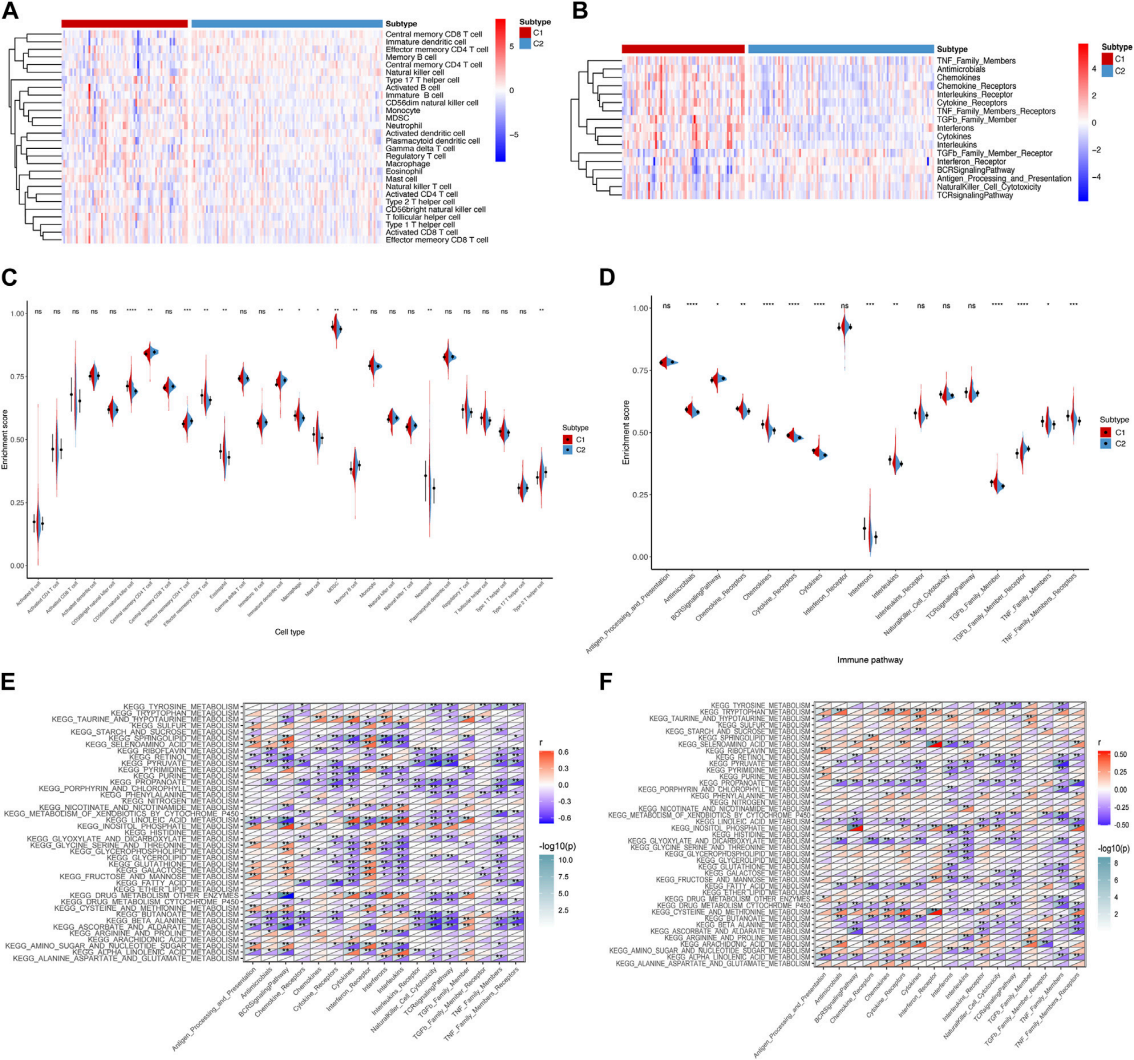
Immune landscape of metabolism-related subtypes in IPF. **(A)** Heatmap and **(C)** violin plots of differential enrichment scores of the immune cell signatures of the two subtypes. **(B)** Heatmap and **(D)** violin plots of differential enrichment scores of immune pathway categories of the two subtypes. Correlation between metabolism-related and immune pathways in **(E)** subtype C1 and **(F)** subtype C2. *p < 0.05, **p < 0.01, ***p < 0.001, ****p < 0.0001; ns, no significance.

Given that immune dysregulation plays a role in IPF (Shenderov et al., 2021), we compared the immune landscapes of the two subtypes. We analyzed immune cell infiltration and the immune pathway enrichment using a heatmap (Figures 2A,B) and a corresponding violin plot (Figures 2C,D). We observed that effector memory CD8^+^ T cells, macrophages, and neutrophils showed increased infiltration in subtype C1 than in C2. Moreover, the antimicrobials, chemokines, cytokines, and transforming growth factor beta (TGF-β) family members were more enriched in subtype C1 than in subtype C2. Based on these findings, we examined the correlation between metabolic and immune pathways to investigate the potential crosstalk in each subtype (Figures 2E,F). We observed more negative correlations between metabolic and immune pathways in subtype C1 than in subtype C2. Collectively, our results suggest that the immune signature differs between the IPF subtypes C1 and C2, indicating differences in the immune microenvironment.

### 3.3 Identification of novel modules and hub genes

**FIGURE 3:**
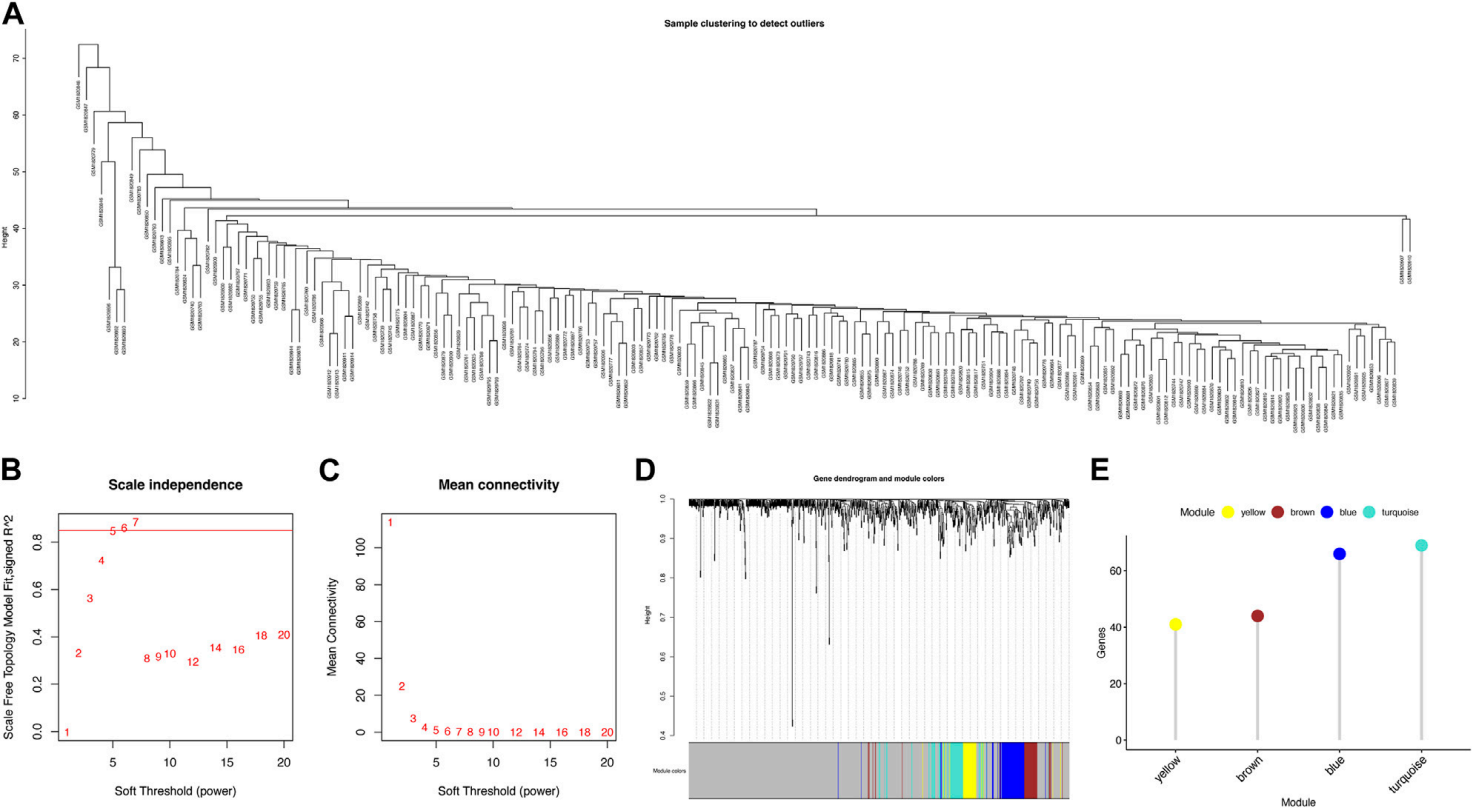
Identification of metabolism-related gene modules. **(A)** Sample clustering. **(B)** Scale-free fit index for various soft-thresholding powers. **(C)** Mean connectivity for various soft-thresholding power values. **(D)** Dendrogram of metabolism-related genes clustered using the one-step method. **(E)** Gene numbers for each module.

**FIGURE 4:**
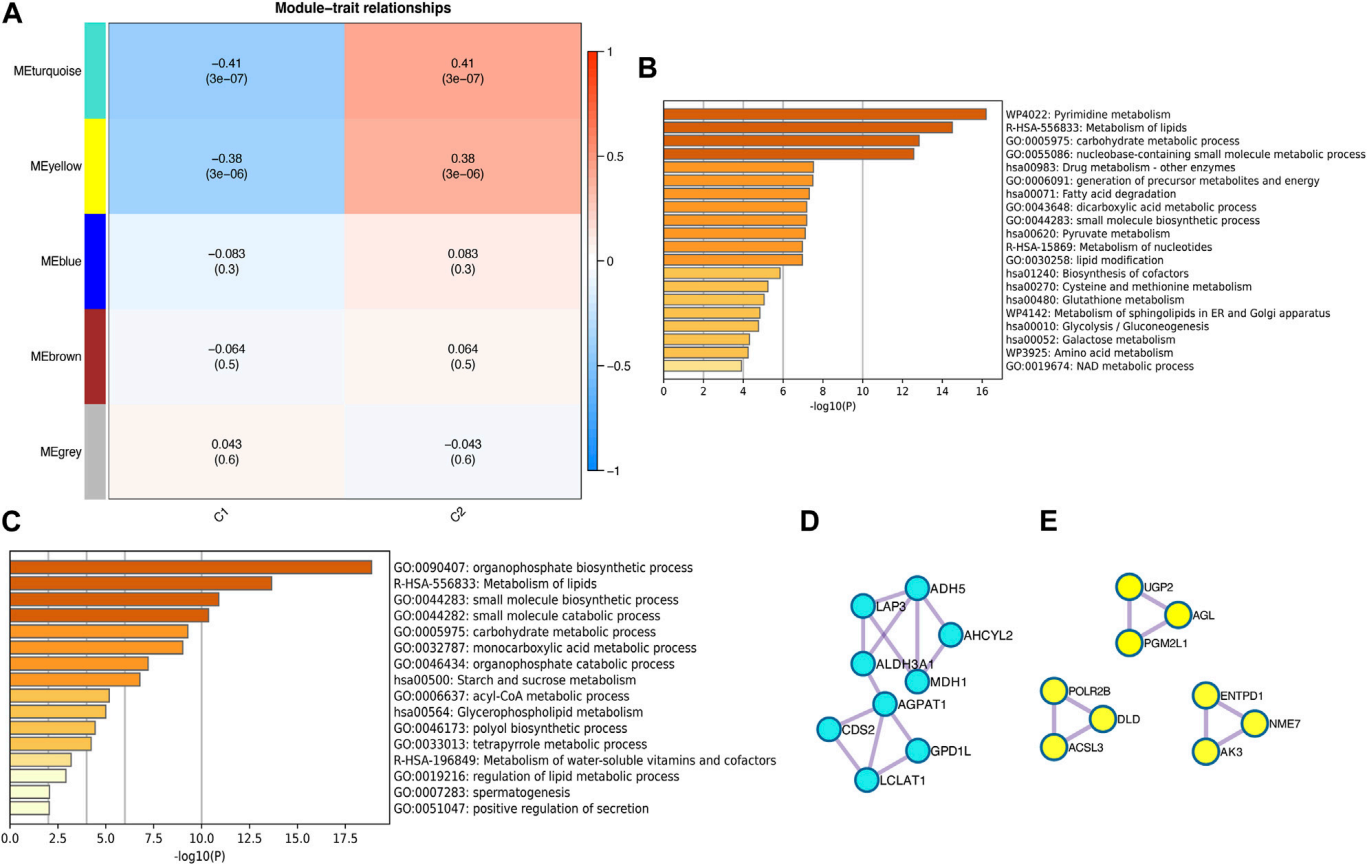
Identification of metabolism-related hub genes in IPF. **(A)** Heatmap of the correlation between modules and metabolism-related subtypes. **(B)** Enrichment analysis of the turquoise module. **(C)** Enrichment analysis of the yellow module. **(D)** Hub genes in the turquoise module. **(E)** Hub genes in the yellow module.

To investigate the key genes in the metabolism-related subtypes, we performed WGCNA. First, we used a hierarchical clustering algorithm to cluster the samples using an average calculation (Figure 3A). To obtain a balance between independence and connectivity, we chose six participants to create a scale-free network (Figures 3B,C) based on which a topological matrix was constructed. We obtained yellow, brown, blue, and turquoise modules, each of which contained clustered genes (Figures 3D,E). To identify the key genes, we first analyzed the module-trait relationships for modules and subtypes (Figure 4A). The turquoise and yellow modules (Supplementary Tables S5 and S6) exhibited apparent differences in their correlations with subtypes. Both were positively correlated with subtype C2 and negatively correlated with subtype C1, indicating that patients with a higher fraction of the two modules were more likely to have subtype C2. The smaller the fraction of the two modules, the more likely it was for the possible subtype to be C1. Notably, both the turquoise and yellow modules were highly enriched in lipid-related metabolism (Figures 4B,C). We then detected the hub genes in each correlated module using the MCODE algorithm (Figures 4D,E). Thus, we identified the hub genes CDS2, LCLAT1, GPD1L, AGPAT1, ALDH3A1, LAP3, ADH5, AHCYL2, and MDH1 in turquoise and PGM2L1, UGP2, AGL, AK3, ENTPD1, NME7, POLR2B, ACSL3, and DLD in yellow. These hub genes might play pivotal roles in the metabolic microenvironment of IPF and could be potential biomarkers for distinguishing the two subtypes of IPF.

### 3.4 Diagnostic efficiency of hub genes

**FIGURE 5:**
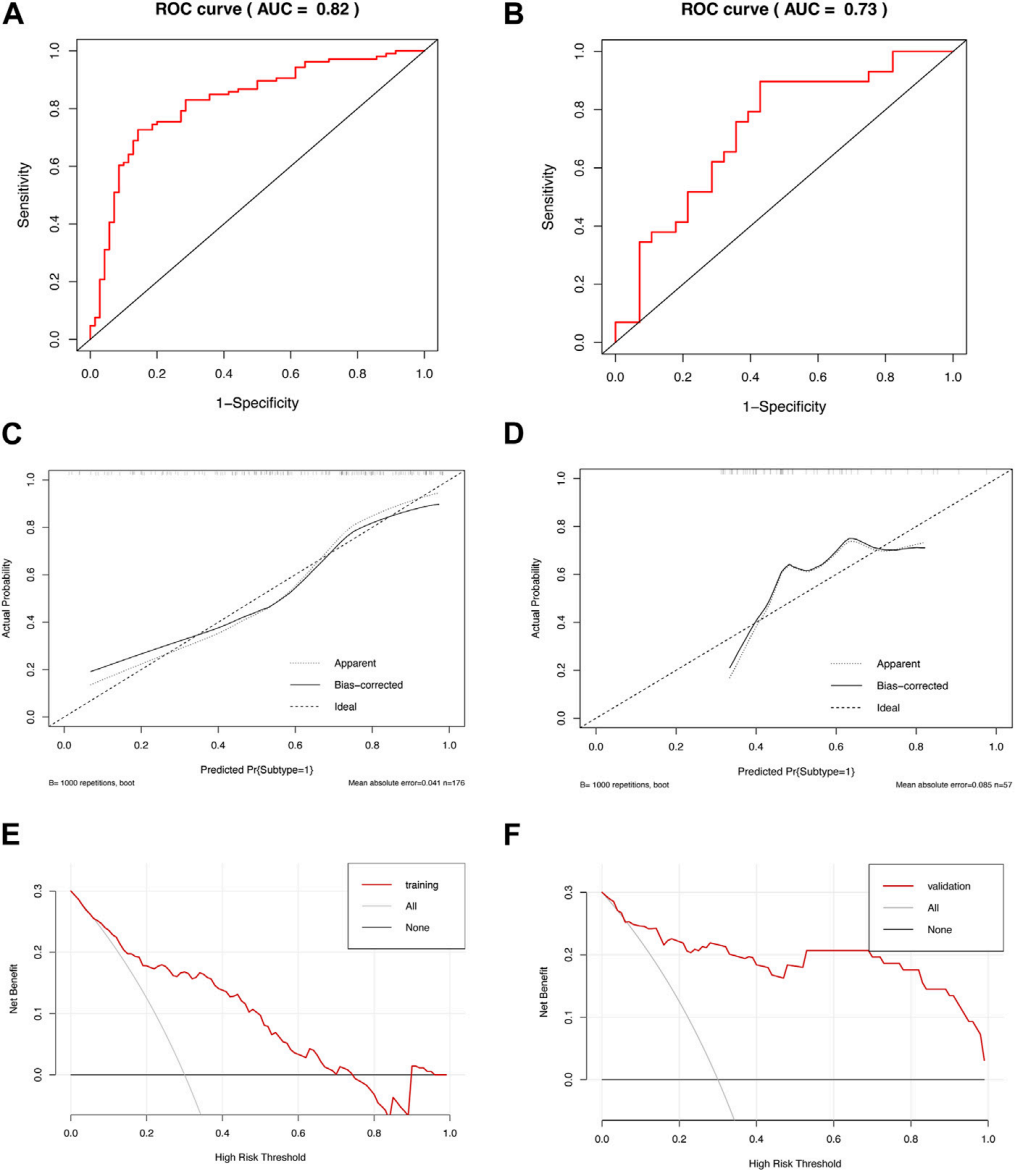
Diagnostic efficiency of the model based on hub genes. ROC curves of the diagnostic model in the **(A)** training and **(B)** validation cohorts. Calibration curves of the diagnostic model in the **(C)** training (HL test, *p* = 0.228) and **(D)** validation cohorts (HL test, *p* = 0.109). DCA of the diagnostic model in the **(E)** training and **(F)** validation cohorts.

We used the identified hub genes to efficiently diagnose the metabolism-related subtypes. To simplify the diagnosis, we selected the hub genes in the turquoise module, which were more closely related, for further analysis. We constructed a diagnostic model based on nine genes in the turquoise module to distinguish between subtypes C1 and C2. We evaluated the discriminatory ability of the model by generating a ROC curve (Figures 5A,B). The AUCs were 0.82 and 0.73 in the training and validation cohorts, respectively, indicating that the model can efficiently discriminate the two subtypes. Additionally, we constructed a calibration curve to evaluate the accuracy of the model (Figures 5C,D). The calibration curves in the training and validation cohorts revealed good predictive accuracy, with a *p*-value >0.05 for HL tests. Furthermore, we performed DCA to analyze the clinical benefits of the model (Figures 5E,F). As shown in the figures, the model could identify the positive benefits of clinical intervention. Collectively, the model constructed using hub genes could be an effective tool for distinguishing the different subtypes of IPF.

### 3.5 Drug probe of different subtypes

**FIGURE 6:**
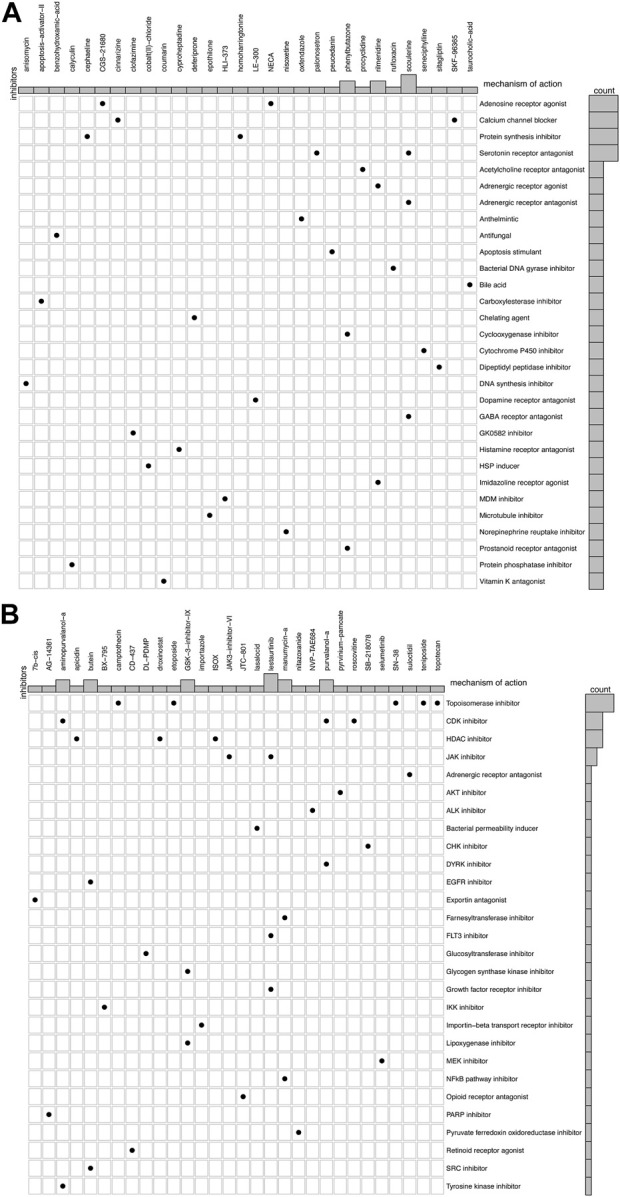
Candidate drugs and their mechanism in different subtypes of IPF. **(A)** 30 compounds and 30 molecular pathways in subtype C1. **(B)** 30 compounds and 28 molecular pathways in subtype C2.

Since there are limited treatment options available for IPF, we screened drug probes for the metabolism-related subtypes using CMap. We identified 30 compounds targeting 30 molecular pathways and 30 compounds targeting 28 pathways for subtypes C1 and C2, respectively (Figures 6A,B). No molecular pathways were shared between the two subtypes for drug probes. Notably, some mechanisms of action involved more than one compound, such as an adenosine receptor agonist and a calcium channel blocker for subtype C1. Conversely, topoisomerase inhibitors exhibited a potent mechanism of action against subtype C2. Therefore, these findings indicate that the drugs suitable for the two subtypes may differ and may require further consideration for future treatment.

## 4 Discussion

In our study, we identified two subtypes based on 41 metabolism-related pathways that showed significant differences in prognosis between the training and validation cohorts. Furthermore, nine genes (CDS2, LCLAT1, GPD1L, AGPAT1, ALDH3A1, LAP3, ADH5, AHCYL2, and MDH1) were investigated to construct a model to distinguish between the two subtypes, with good discrimination and calibration. Notably, subtype C1, which displayed a low metabolic level, demonstrated a shorter survival time than subtype C2, which exhibited a high metabolic level. These findings suggest that metabolism-related pathways may help predict the prognosis of patients with IPF. To our knowledge, this is the first study to classify patients with IPF based on multiple metabolic pathways and screen potential treatments for each subtype.

Derricks et al. (2013) demonstrated that metabolic agents such as ascorbate could upregulate the synthesis of elastin and collagen, which could promote the deposition of the extracellular matrix (ECM). Lipid metabolism is a critical component in this process. In a mouse model, a lack of lipid synthesis increased endoplasmic reticular stress, which aggravated the remodeling of lung tissue (Romero et al., 2018). Similarly, dysfunction of apolipoprotein A could result in cholesterol deposition in alveolar macrophages, leading to the formation of foam cells, fibrosis, and remodeling of the lung tissue (Wygrecka et al., 2023). Ivanova et al. (2013) demonstrated promising results that liposomal prostaglandin E2 attenuated the extent of bleomycin-induced fibrosis in mice. Moreover, in clinical research, lipid metabolism-related products, such as amyloid A and adiponectin, are prognostic markers in clinical research (Vietri et al., 2019; d’Alessandro et al., 2020). Additionally, the activation of hypoxic response elements can modify glycolysis to promote the proliferation and differentiation of myofibroblasts during IPF progression (Dabral et al., 2019; Contreras-Lopez et al., 2020). Overall, subtyping based on metabolism-related pathways is an effective way of determining the prognosis of patients with IPF.

To determine the differences between the two subtypes, we performed an immune-related analysis. There was a negative correlation between metabolism and immunity in subtype C1. Owing to the low metabolism level in subtype C1, it may reach a more pronounced immune signature than subtype C2. This was demonstrated by the significant infiltration of immune cells and enrichment of immune pathways in subtype C1. Therefore, we hypothesized that metabolic dysregulation might correlate with immune status and promote pulmonary fibrosis by causing aberrative activation of immune cells. Researchers have previously used single-cell sequencing to identify an increase in CD8 effector memory T cells and alveolar macrophages with interferon-gamma (IFN-γ) enrichment in lung tissues affected by IPF, indicating the activation of adaptive immunity (Serezani et al., 2022). Additionally, monocyte-derived alveolar macrophages reportedly promote fibrosis, indicating the heterogeneity of macrophages in the IPF (Misharin et al., 2017). Furthermore, metabolic reprogramming in macrophages has been extensively studied, which contributes to the metabolic dysregulation of cholesterol and fatty acids (Batista-Gonzalez et al., 2019; Ahangari et al., 2023). Cholesterol deposition in macrophages results in the formation of foam cells. The accumulation of these leads to the activation of inflammatory response and increased oxidative stress in lung tissue, thereby promoting fibroblast differentiation and collagen deposition; this contributes to the progression of IPF (Wygrecka et al., 2023). The utilization and oxidation of fatty acids can provide energy for M2 macrophages to perform their biological functions, promoting fibrosis via TGF-β in IPF (Odegaard and Chawla, 2011; He et al., 2016; Rui et al., 2022). Meanwhile, a previous study found that M2 cells have glucose utilization properties, which can activate M2 polarization (Huang et al., 2016). Therefore, the aberrant metabolism-related pathway activation promotes fibrotic functions in macrophages. Neutrophils release neutrophil extracellular traps (NETs), which play a pro-fibrotic role in response to chronic inflammatory stimulation by activating the proliferation and differentiation of fibroblasts to promote collagen deposition (Chrysanthopoulou et al., 2014; Suzuki et al., 2020). Furthermore, mitochondrial inner membrane proteins, which participate in mitochondrial energy metabolism and maintain the stability of metabolic pathways, are essential for forming and releasing extracellular trapping nets from neutrophils (Amini et al., 2018). Thus, the relationship between metabolism and immunity may result in a high level of immunity in subtype C1, leading to the progression of pulmonary fibrosis and a poor prognosis.

We constructed a nine-gene diagnostic model to efficiently distinguish between the two subtypes. One of the genes in this model, CDS2, is involved in synthesizing phosphatidylinositol and plays a novel role in the progression of inflammation and fibrosis via mitochondrial dysfunction (Xu et al., 2022). Similarly, LCLAT1, which regulates linoleic acid levels, affects mitochondrial function and reactive oxygen species (ROS) generation, contributing to pulmonary fibrosis (Huang et al., 2014). Another gene, GPD1L, is involved in fibroblast proliferation and collagen synthesis in the atrium (Hao et al., 2022). Other genes, including AGPAT1, ALDH3A1, LAP3, and ADH5, have been shown to have different functions in fibrosis in various diseases (Tang et al., 2013; Niu et al., 2019; Irungbam et al., 2020; Talpan et al., 2023). Notably, we identified the novel role of AHCYL2 and MDH1 in IPF; however, further validation is required.

The nine-gene model demonstrated statistically robust results, with significant discrimination and calibration in both the training and validation cohorts, suggesting its potential clinical value. Furthermore, a drug probe was developed to distinguish between the two subtypes. It utilizes the gene expression signatures in different subtypes to connect with similar expression signatures in CMap, suggesting that the corresponding drugs that had caused these CMap signatures may confer related biological effects. Therefore, in this study, the corresponding CMap drug was the potential drug we explored for treating each IPF subtype. Drugs for subtype C1 focus on adenosine receptor activation, which can inhibit macrophage profibrotic function and abnormal metabolic activation (Csoka et al., 2014). Drugs for subtype C2, such as those targeting the inhibition of CDK, affect glucose and lactic acid metabolism, which might be treatable for patients (Carvalhal et al., 2003). The drugs and mechanisms of action in the two subtypes suggest that different metabolism-related subtypes might have different treatable traits.

Our study has several limitations. First, the subtyping validation was performed in only one cohort, which might limit the generalizability of the results. Therefore, further validation using larger cohorts is required to increase the reliability of the results. Second, the lack of experimental verification in this study limits our understanding of the metabolic differences between the two subtypes. Thus, future studies should focus on verifying the metabolic levels in animal models and evaluating immune cell infiltration *in vivo* to further understand the biological mechanisms underlying IPF. Third, our study only identified markers for subtyping IPF; their efficiency in clinical practice still needs to be tested in human subjects. Therefore, further studies are needed to examine the efficiency of these markers and their value in clinical practice.

## 5 Conclusion

In conclusion, our findings confirmed that metabolic heterogeneity exists in patients with IPF. Furthermore, metabolic dysregulation contributes to the progression of pulmonary fibrosis, and different metabolic levels could result in different prognoses. Our diagnostic model, with two IPF prognosis-related subtypes based on 41 metabolism-related pathways, can aid clinicians in efficiently identifying patients with a poor prognosis, thereby facilitating individualized therapeutic management and shorter follow-up periods. Furthermore, the potential drugs identified in our study could aid in treating patients based on their distinctive subtypes. Further investigation is required to validate the clinical efficacy of the identified drugs and their potential to target metabolic pathways and treat IPF. Nonetheless, our findings provide novel insights into distinguishing patients with IPF based on metabolism-related pathways and developing individual treatment strategies for patients in different subtypes.

## Data Availability

The original contributions presented in the study are included in the article/Supplementary Material; further inquiries can be directed to the corresponding authors.
